# Fine needle aspiration biopsy of the liver: Algorithmic approach and current issues in the diagnosis of hepatocellular carcinoma

**DOI:** 10.1186/1742-6413-2-7

**Published:** 2005-06-08

**Authors:** Aileen Wee

**Affiliations:** 1 Professor and Senior Consultant, Department of Pathology, National University of Singapore, National University Hospital, 5 Lower Kent Ridge Road, Singapore, 119074, Republic of Singapore

**Keywords:** Diagnostic algorithm, fine needle aspiration biopsy, hepatocellular carcinoma, immunohistochemistry, liver

## Abstract

The role of fine needle aspiration biopsy (FNAB) in the evaluation of focal liver lesions has evolved. Guided FNAB is still useful to procure a tissue diagnosis if clinical, biochemical and radiologic findings are inconclusive. Major diagnostic issues include: (i) Distinction of benign hepatocellular nodular lesions from reactive hepatocytes, (ii) Distinction of well-differentiated hepatocellular carcinoma (WD-HCC) from benign hepatocellular nodular lesions, (iii) Distinction of poorly differentiated HCC from cholangiocarcinoma and metastatic carcinomas, (iv) Determination of histogenesis of malignant tumor, and (v) Determination of primary site of origin of malignant tumor. This review gives a general overview of hepatic FNAB; outlines an algorithmic approach to cytodiagnosis with emphasis on HCC, its variants and their mimics; and addresses current diagnostic issues. Close radiologic surveillance of high-risk cirrhotic patients has resulted in the increasing detection of smaller lesions with many subjected to biopsy for tissue characterization. The need for tissue confirmation in clinically obvious HCC is questioned due to risk of malignant seeding. When a biopsy is indicated, core needle biopsy is favored over FNAB. The inherent difficulty of distinguishing small/early HCC from benign hepatocellular nodular lesions has resulted in indeterminate reports. Changing concepts in the understanding of the biological behavior and morphologic evolution of HCC and its precursors; and the current lack of agreement on the morphologic criteria for distinguishing high-grade dysplastic lesions (with small cell change) from WD-HCC, have profound impact on nomenclature, cytohistologic interpretation and management. Optimization of hepatic FNAB to enhance the yield and accuracy of diagnoses requires close clinicopathologic correlation; combined cytohistologic approach; judicious use of ancillary tests; and skilled healthcare teams.

## Review

Until recently, guided fine needle aspiration biopsy (FNAB) was accepted as a safe procedure to procure tissue diagnosis in the management of patients with focal liver lesions. However, the role of FNAB in the evaluation of such lesions, especially hepatocellular carcinoma (HCC), and the diagnostic challenges it poses have evolved [1-4]. Much diagnostic information can be gleaned nowadays from an ever-expanding array of tumor markers and sophisticated imaging modalities. This has obviated the need, in some practices, for tissue confirmation in clinically obvious HCC. Surveillance of high-risk cirrhotic patients, has contributed to an increasing detection of focal liver lesions that are <2 cm in diameter [5,6]. Study of autopsy material and cirrhotic explants, coupled with immunohistochemical and/other ancillary techniques, have led to better understanding of the morphologic evolution and biological behavior of certain hepatocellular nodular lesions [7-10]. This changing concept in the evolution of HCC and its precursor lesions has great impact on nomenclature [11], cytohistologic interpretation, management strategies and treatment outcomes [5,12]. Critical refinement of current histopathologic criteria for the diagnosis of small ("early") HCC is necessary with increasing demands for tissue characterization of small "suspicious" nodules [13-17]. The window of opportunity in the treatment of HCC is limited as patients tend to present with advanced cancers.

The review gives an algorithmic approach to the FNAB diagnosis of focal liver lesions; an overview of current diagnostic issues concerning hepatic FNAB; problems and pitfalls in the cytodiagnosis of well-differentiated hepatocellular nodular lesions; and diagnostic utility of immunohistochemistry.

## General approach to focal liver lesions

Focal lesions in the liver range from cysts and inflammatory processes to neoplasms, be they benign or malignant, primary or metastatic [11,18]. This realm of pathology comes with its share of diagnostic challenges and pitfalls. Although imaging and tumor markers can provide a diagnosis in many instances, tissue confirmation may be warranted under circumstances where the clinical, biochemical and imaging profiles are not conclusive [19]. A combined smearing and microhistology approach is strongly recommended [20-24]. Smears are air-dried and stained with Diff-Quik/May-Grunwald-Giemsa as well as fixed in 95% alcohol and stained by the Papanicolaou method. Particulate material is formalin-fixed for preparation of cell blocks for histologic study and for special and immunostains. Use of FNAB needles (21 gauge) with cutting mechanism may provide microbiopsy tissue cores.

Apart from cystic/inflammatory conditions, the major diagnostic issues are: (i) Distinction of benign hepatocellular nodular lesions, namely, macroregenerative nodule (MRN), dysplastic nodule (DN), focal nodular hyperplasia (FNH) and liver cell adenoma (LCA), from reactive hepatocytes; (ii) Distinction of well-differentiated HCC (WD-HCC) from benign hepatocellular nodular lesions; (iii) Distinction of poorly differentiated HCC (PD-HCC) from cholangiocarcinoma (CC) and metastatic carcinomas; (iv) Determination of histogenesis of malignant tumor; and (v) Determination of primary site of origin of malignant tumor.

## Algorithmic approach

A diagnostic algorithm for FNAB diagnosis of focal liver lesions is given in Table 1. The emphasis is on HCC, its variants, and their differentiation from benign and malignant mimics [20].

**Table 1:** Diagnostic algorithm for fine needle aspiration cytology of the liver

***Patient with liver mass/es***
STEP 1: Establish category of clinical presentation

***Imaging***
STEP 2: Establish category of radiologic findings

***Fine needle aspiration biopsy***
STEP 3: Establish nature of cytohistologic findings

***Ancillary techniques***
*(special stains, immunohistochemistry)*
STEP 4: Further confirm nature of cytohistologic findings

***Clinicopathologic correlation***
STEP 5: Establish final diagnosis based on multidisciplinary approach

### Step 1: Establish category of clinical presentation

A patient may present with a liver mass/es under the following clinical scenarios: (i) Routine medical check-up, (ii) Chronic liver disease due to, for example, hepatitis B or C virus infection, and alcoholism, (iii) Known cancer cases, (iv) Symptomatic patients, and (v) In childhood. Important relevant data include serum alpha-fetoprotein (AFP) and carcinoembryonic antigen (CEA) levels, hepatitis virus markers, results of liver function tests, presence of cirrhosis, biliary tract disease, calculi, liver fluke infestation, and history of hormone usage. Radiologic correlation is mandatory.

### Step 2: Establish category of radiologic findings

In many instances, a preoperative diagnosis can be achieved with a high degree of accuracy based on non-invasive imaging techniques and close clinical correlation. FNAB is useful in defining those lesions without characteristic imaging appearance. The solid or cystic nature of the lesion; number, size and location of the lesion/s; absence or presence of hepatomegaly, cirrhosis, steatosis, regional lymphadenopathy and calculi; and status of the biliary tract are important clues to the final diagnosis. Imaging of liver masses can be divided into two categories, namely, cystic and solid lesions. The focus is on a rational and pragmatic approach to hepatic FNAB. A list of entities is given in Table 2 as a working guide.

**Table 2:** Cystic and Solid Lesions of the Liver

***CYSTIC LESIONS***
**BENIGN AND MALIGNANT ENTITIES**
Congenital cysts
Parasitic cysts: *Hydatid cysts*
Abscesses: *Pyogenic, amebic and fungal abscesses*
Granulomas
Inflammatory pseudotumors (can be solid)
Cystic neoplasms: *Biliary cystadenoma/cystadenocarcinoma, cholangiocarcinoma associated with cystic liver disease, cystic metastases (from ovary, pancreas)*

***SOLID LESIONS***
**BENIGN ENTITIES**
**Benign hepatocellular nodular lesions**
Regenerative nodules (cirrhosis, nodular regenerative hyperplasia)
Macroregenerative nodule
Dysplastic nodule
Focal nodular hyperplasia
Liver cell adenoma
Focal fatty change

**Benign non-hepatocellular nodular lesions**
Bile duct adenoma, hamartoma
Hemangioma
Angiomyolipoma
Extramedullary hematopioesis

**MALIGNANT ENTITIES**
**Classic primary liver cancers**
Hepatocellular carcinoma (HCC)
Intrahepatic cholangiocarcinoma

**Variants of primary liver cancers**

***Variants of hepatocellular carcinoma:***
HCC with fatty change
HCC, clear cell type
HCC, small cell type
HCC, undifferentiated type
HCC, spindle cell type
HCC, giant cell type
Fibrolamellar HCC
HCC with biliary differentiation
Combined hepatocellular-cholangiocarcinoma

***Variants of cholangiocarcinoma:***
Biliary papillary neoplasia / Intraductal papillary cholangiocarcinoma
Mucinous intrahepatic cholangiocarcinoma

**Mimics of primary liver cancers**
Adenocarcinoma
Squamous cell carcinoma
Small round cell malignancy
Clear cell malignancy
Pleomorphic cell malignancy
Spindle cell malignancy
Giant cell malignancy
Hepatoid carcinoma; alpha-fetoprotein-producing carcinoma

### Step 3: Establish nature of cytohistologic findings

#### FNAB of normal/reactive liver

**Figure 1:**
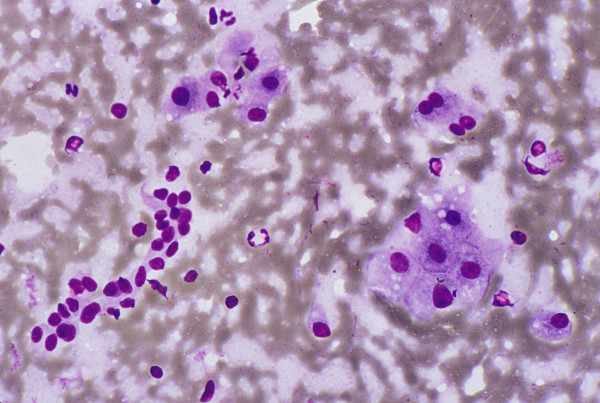
***Normal and reactive hepatocytes: FNAB***. Monolayered clusters of loosely-cohesive, well-defined, polygonal cells with ample dense, granular cytoplasm, round central nuclei and low nuclear-cytoplasmic ratio. Note the double-layered row of ductular epithelial cells with scanty cytoplasm (May-Grunwald-Giemsa).

**Figure 2:**
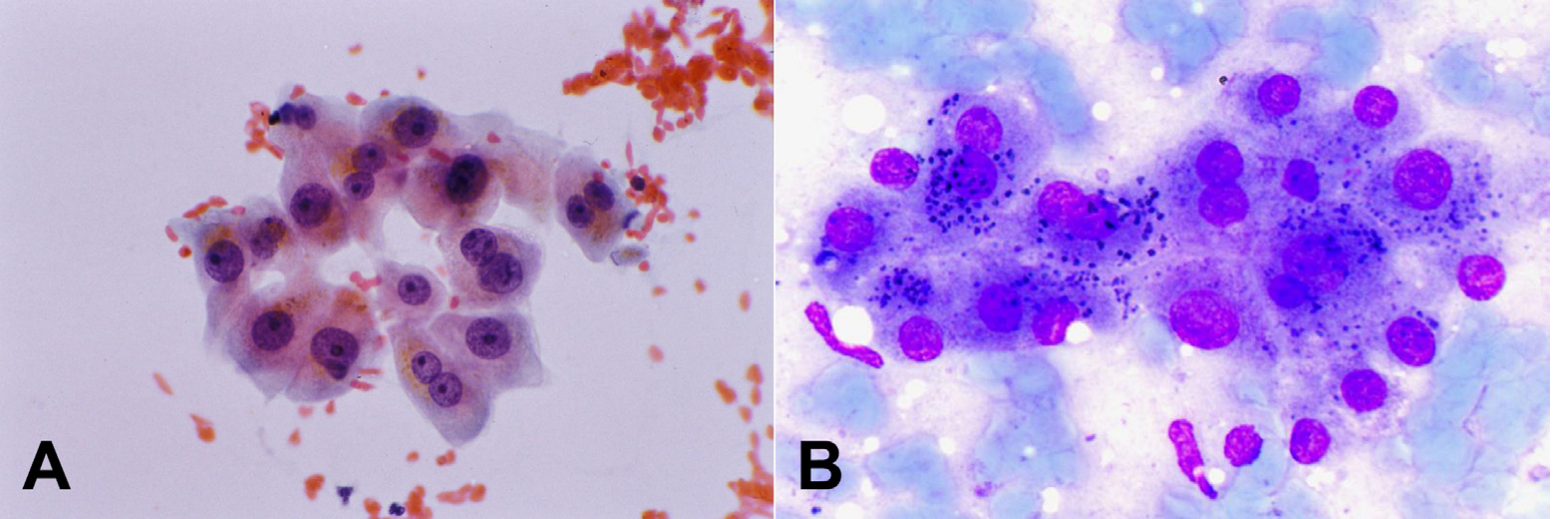
***Normal and reactive hepatocytes: FNAB***. (A) Hepatocytes show dense granular cytoplasm, round central nuclei, well-delineated nuclear membrane, distinct nucleoli, granular chromatin and binucleation. Note polymorphism displayed by nonneoplastic hepatocytes. The cells contain brown granules of lipofuscin pigment in the cytoplasm (Papanicolaou). (B) Lipofuscin appears as black granules. The two elongated nuclei are likely to be Kupffer cells (May-Grunwald-Giemsa).

The liver parenchyma comprises a heterogeneous population of hepatobiliary and related cells, namely, hepatocytes, bile duct and ductular epithelia (Figure 1); and Kupffer, endothelial, mesothelial and inflammatory cells. Hepatocytes often contain intracytoplasmic inclusions, such as, fat vacuoles, Mallory bodies and to a lesser extent, hyaline bodies; as well as intranuclear cytoplasmic inclusions. Pigments, such as lipofuscin (Figure 2), bile and iron, may be present.

#### FNAB of liver with large and small cell change

**Figure 3:**
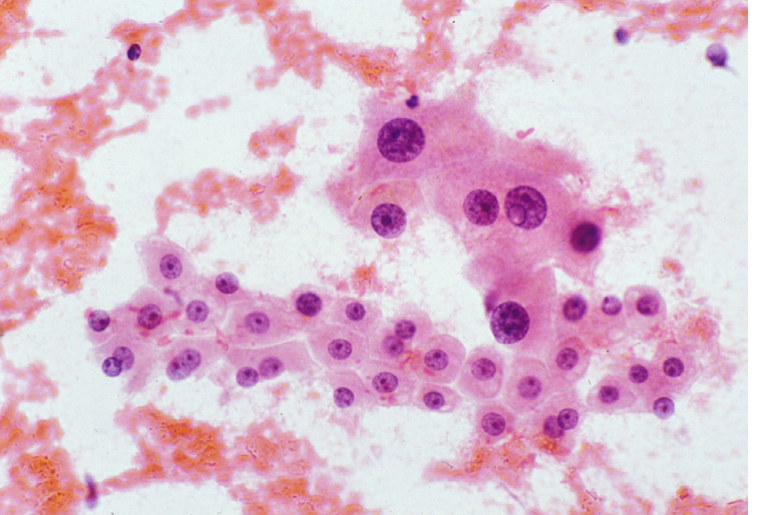
***Hepatocytes with large cell change: FNAB***. There is simultaneous nuclear and cell enlargement of the hepatocytes, thus maintaining the nuclear-cytoplasmic ratio of 1/3. Note mild nuclear atypia (Papanicolaou).

The terms "large cell change" and "small cell change" have replaced large and small cell dysplasia [18]. Hepatocytes with large cell change, considered a low-grade lesion, exhibit both cell and nuclear enlargement with nuclear atypia but retaining the normal nuclear-cytoplasmic ratio (N/C) of ≤ 1/3, by eyeballing the diameters of the nucleus and the cell (Figure 3). On the other hand, in small cell change, considered a high-grade lesion and putative precancerous link with HCC, the hepatocytes are small and monotonous with subtle increase in N/C ratio; they impart an impression of nuclear crowding. Both types of changes are common in cirrhosis. The atypical cells can form dysplastic foci (<1 mm diameter) or nodules (>1 mm diameter) in both cirrhotic and non-cirrhotic livers [18].

#### FNAB of a liver mass: Stepwise approach

Liver aspirates can come from malignant or benign conditions of hepatocellular or non-hepatocellular origin [25-27]. Certain entities can give characteristic gross appearances when smeared [20]. Naked eye inspection coupled with low power scanning view of the smears is a helpful adjunctive step to determine: (i) Patterns of spread of smears, (ii) Malignant or benign cell picture, (iii) Hepatocytic nature or otherwise, and (iv) Monotonous hepatocytic population or heterogeneous liver parenchymal components.

##### Patterns of spread of smears

**Figure 4:**
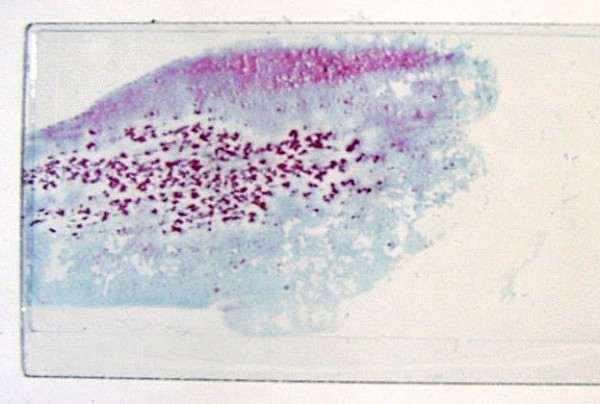
***Naked eye inspection of hepatic aspirate***. Uniformly granular pattern of spread of classic hepatocellular carcinoma. Note the regularly irregular tumor fragments which tend to be equidistant (Papanicolaou).

A. Uniformly granular pattern. Hypercellular. Regularly irregular tissue fragments; evenly distributed in rows with tendency to be equidistant. Most likely HCC (Figure 4).

B. Non-uniformly granular pattern. Hypercellular. A mixed picture of irregular tissue fragments of variable size/shape; may include uniform granules. Variable cell picture -malignant to benign lesions of any origin.

C. Scanty pattern. Hypocellular. Streaks or fine clumps of cells. Variable cell picture -desmoplastic malignancies or benign conditions, such as cyst contents and hepatocellular nodular lesions. Pitfalls: Necrotic or cystic neoplasm; non-lesional sample.

D. Fluid pattern. Amorphous look. Thin fluid or thick pus. Usually benign and non-hepatocellular, such as abscess or infected cyst contents. Pitfalls: Dissociated tumor cells, such as lymphomas.

E. Microbiopsy pattern. Short and narrow, difficult-to-focus tissue cores, usually composed of nonneoplastic liver parenchyma with intact architecture. Admixed with other patterns, if the specimen is representative and includes lesional tissue. The clue is from the other patterns. Rarely, tumor tissue.

#### FNAB of hepatocellular carcinoma

HCC can be small/focal, solitary/large, and multifocal/diffuse; with satellite nodules and large vein involvement. Classic HCC is usually graded into well, moderately or poorly differentiated lesions. Histologic patterns comprise trabecular-sinusoidal, pseudoacinar and solid types; combinations are frequent [28]. Close attention should be paid to architectural details in cell blocks/microbiopsies and smears. Accurate distinction from metastases, especially unresectable lesions, is necessary for appropriate therapy. One should be aware that there are limitations to the cytodiagnosis of HCC [23,29-31].

##### Smears

**Figure 5:**
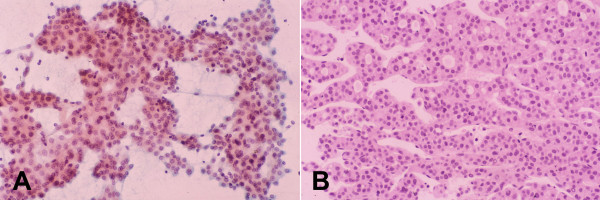
***Well-differentiated hepatocellular carcinoma with trabecular and pseudoacinar patterns: FNAB***. (A) Thick arborizing cords of malignant hepatocytes showing cellular monotony, increased nuclear-cytoplasmic ratio, and impression of nuclear crowding. The circular spaces among the cords represent pseudoacini (Papanicolaou). (B) Corresponding histologic section of the tumor shows trabecular-sinusoidal arrangement with pseudoacini. Note the uniformity of the tumor cells and cords 2 to 3 cells thick (H&E).

**Figure 6:**
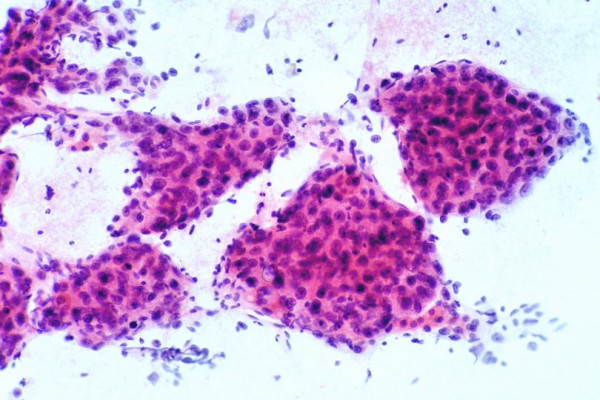
***Moderately differentiated hepatocellular carcinoma: FNAB***. Thick cords of malignant hepatocytes are wrapped by peripheral endothelium. They appear to be floating on transverse section view (Papanicolaou).

**Figure 7:**
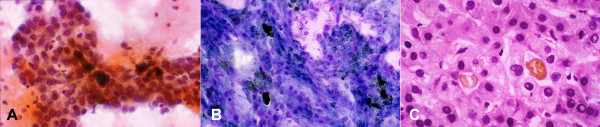
***Moderately differentiated hepatocellular carcinoma with pseudoacinar pattern: FNAB***. (A) Pseudoacini filled with bile which appears as dark brown blobs (Papanicolaou) (B) Bile appears black (May-Grunwald-Giemsa). (C) Corresponding histologic section of the tumor shows cystically dilated canaliculi filled with golden-brown bile and surrounded by polygonal cells with central nuclei (H&E).

**Figure 8:**
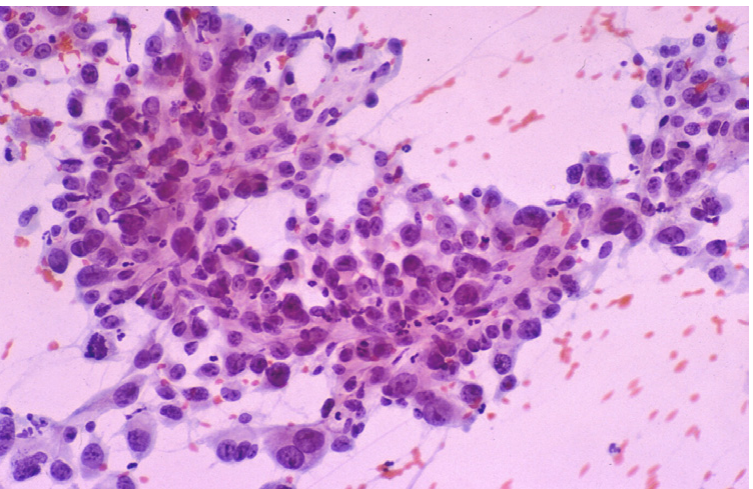
***Poorly differentiated hepatocellular carcinoma: FNAB***. High-grade tumor shows marked pleomorphism but still retaining some hepatocytic characteristics (Papanicolaou).

**Figure 9:**
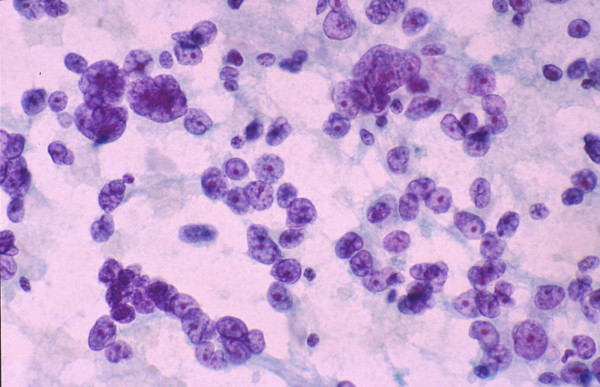
***Poorly differentiated hepatocellular carcinoma: FNAB***. Atypical naked hepatocytic nuclei exhibit pleomorphism, thin nuclear membrane, nuclear contour irregularities, prominent nucleoli and multinucleation (Papanicolaou).

• Hypercellular smears with uniformly granular pattern of spread of the cells.

• Cohesive clusters of malignant hepatocytes with arborizing, tongue-like projections of broad cords (>2 cells thick) that may be wrapped by peripheral endothelium (Figures 5, 6).

• Rows of transgressing endothelium in larger aggregates; basement membrane material ("sinusoidal capillarization") best seen in Giemsa preparations.

• Cohesion is the rule; however, tendency to dissociation noted in highly WD-HCC and PD-HCC.

• Pseudoacini containing bile or pale secretions are not uncommon. The spaces are surrounded by polygonal cells with central nuclei similar to adjacent cells (Figure 7).

• Hepatocytic characteristics include polygonal cells with well-defined borders, ample granular cytoplasm, central round nucleus, well-delineated nuclear membrane, prominent nucleolus and fine, irregularly granular chromatin. Mitoses increase with nuclear grade. Cytologic features of malignancy are wanting at the highly WD-HCC end whereas resemblance to hepatocyes is lacking at the PD-HCC end.

• Tumor cells may be smaller, larger or of the same size as normal hepatocytes. WD-HCC cells tend to be conspicuous by their small size, monotony, subtle increase in N/C ratio and nuclear crowding. PD-HCC cells tend to be pleomorphic (Figure 8).

• Atypical naked hepatocytic nuclei may abound (Figure 9).

• Multinucleated tumor giant cells may be of "osteoclastic" or pleomorphic type. The former shows nuclear features akin to adjacent HCC cells. Tumor giant cells may be found in all grades of HCC. Their presence does not necessarily upgrade the tumor.

• Bile may be present within tumor cells or in canaliculi or pseudoacini.

• Intracytoplasmic fat and glycogen vacuoles are common. Intracytoplasmic inclusions include hyaline, pale and Mallory bodies. Intranuclear cytoplasmic inclusions are not specific.

• Bile duct epithelial cells, if present, are few and far apart. Kupffer cells may be seen.

##### Cell blocks/microbiopsies

• Microhistology provides additional invaluable architectural details such as trabecular-sinusoidal pattern formed by broad trabeculae (>2 cells thick); pseudoacini; unpaired arteries; and deficient/virtually absent reticulin framework.

#### FNAB of well-differentiated hepatocellular nodular lesions

The accuracy of cytodiagnosis at this end of the spectrum is often an issue with indeterminate reports being rendered [14,32]. The diagnostic dilemmas are: (i) Is the sample representative? (ii) Are the hepatocytes malignant (**highly WD-HCC**) or benign? (iii) If benign, are they neoplastic (**LCA**) or nonneoplastic hepatocytes? (iv) If nonneoplastic, are they intralesional (**FNH, DN or MRN**) or extralesional hepatocytes (**cirrhosis **or **normal liver**), with/without **fatty change**? Cytologic features predictive of HCC include increased N/C ratio, cellular monomorphism, nuclear crowding, trabeculae >2 cells thick, atypical naked hepatocytic nuclei and lack of bile duct cells [23,33]. Cytologic parameters distinguishing highly WD-HCC from cirrhosis include well-defined cytoplasmic borders, scant cytoplasm, monotonous cytoplasm, thick cytoplasm, eccentric nuclei and increased N/C ratio [34].

#### FNAB of variants of hepatocellular carcinoma

HCC is well known for its histologic variations and subtypes. A large tumor can harbor areas that are more easily recognizable than others. This has significant practical implications on the number of passes and sampling in hepatic FNAB.

**Figure 10:**
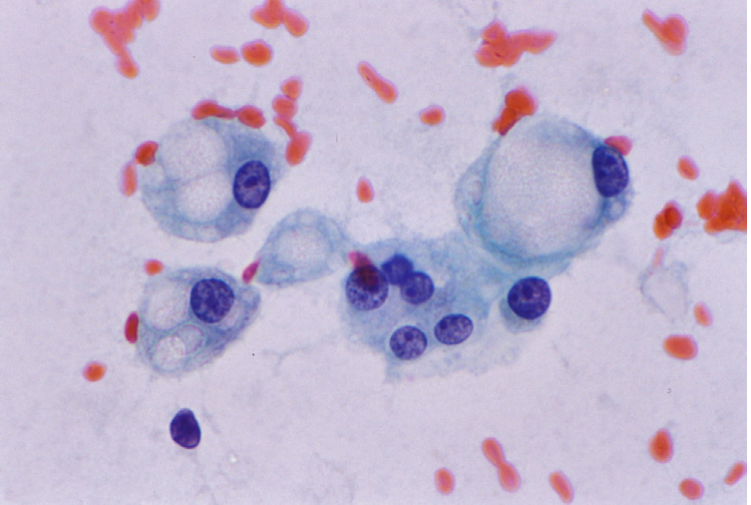
***Fatty change: FNAB***. Hepatocytes exhibit polymorphism and contain cytoplasmic fat vacuoles of varying sizes pushing nucleus to one side. Cytologic picture can mimic signet-ring cell adenocarcinoma (Papanicolaou).

The variants of HCC include:

• **HCC with fatty change**: Fatty change can occur in HCC without associated steatosis. Small (early) lesions are prone to fatty change due to inadequate vascularization. WD-HCC cells with cytoplasmic fat vacuoles can be mistaken for hepatocytes from fatty liver or focal fatty change (Figure 10) [35,36]. PD-HCC cells with fat vacuoles may mimic malignant lipoblasts or signet-ring adenocarcinoma cells.

• **HCC, clear cell type: **Clear cell change in hepatocytes is due to abundant cytoplasmic glycogen or lipid content [37]. Tumor cells with empty-looking vacuoles after removal of glycogen during processing may mimic fatty change. Focal clear cell change is frequent. Diffuse clear cell change occurs in <10% of cases of HCC [38].

• **HCC, small cell type: **This type is reminiscent of neuroendocrine tumors with tendency to dissociation and microacinar formation but no obvious trabecular pattern [39,40]. Scrutiny of the nuclear/chromatin features should reveal the true histogenesis of the small round cells.

• **HCC, undifferentiated type**: The tumor cells are larger than the small cell category with no obvious hepatocytic characteristics.

• **HCC, spindle cell type: **This is rare and is more likely to be seen with tumor giant cells as part of a larger tumor [41].

• **HCC, giant cell type: **This pure variant is rare.

• **Fibrolamellar HCC: **This occurs in non-cirrhotic livers of young patients and has a good prognosis. It comprises large, discohesive polygonal hepatocytes with abundant oncocytic cytoplasm and lamellar fibrosis. Pale bodies are common [42,43].

• **HCC with biliary differentiation**: Some HCC are positive for biliary markers (AE1/3, CK19) [44]. The stem cell theory with bipotential progenitor cells capable of developing into either hepatocytes or biliary epithelial cells provides a satisfactory explanation for primary liver cancers arising from different stages of the cell lineage [45].

• **Combined hepatocellular-cholangiocarcinoma (CHCC-CC): **This is a rare tumor containing unequivocal elements of HCC and CC that are intimately admixed with a transitional component [46,47]. The HCC cells are expected to be AFP and Hep Par 1-positive and show polyclonal CEA (*p*CEA) canalicular staining. The CC cells are AE1/3-positive and show brush border/diffuse cytoplasmic *p*CEA reactivity. The intermediate cells exhibit hybrid features with equivocal immunoprofiles.

#### FNAB of cholangiocarcinoma and its variants

Intrahepatic CC are rare and usually occur in non-cirrhotic livers in close proximity with sizeable bile ducts. Predisposing diseases include clonorchiasis, opisthorchiasis, hepatolithiasis and primary sclerosing cholangitis. CC are usually well to moderately differentiated adenocarcinomas with variable degree of desmoplasia. They can be categorized into papillary and/or tubular adenocarcinomas. Mucin production is minimal. A squamous component may be present. Adjacent biliary epithelial changes include carcinoma-in-situ and biliary ductular proliferation. There are three cytologic smear patterns, namely (i) Scanty cell smear pattern, (ii) Adenocarcinoma with proliferating ductular clusters, and (iii) Adenocarcinoma without prominent ductular clusters [20].

The variants of CC include:

• Biliary papillary neoplasia / intraductal papillary CC [48]

• Mucinous intrahepatic CC [20]

#### FNAB of non-hepatocellular carcinoma malignancies

That the liver is a common target for metastases makes the separation between primary and secondary malignancies all the more difficult, especially when the particular histologic subtype can arise in the liver as well. Categorizing them based on cytologic patterns is a good start.

• **Adenocarcinoma: **Most are metastases from stomach, colorectum, pancreas, breast and lungs. Colorectal metastases have much tumor diathesis. Signet-ring cell adenocarcinomas are likely to be gastric in origin. Pancreaticobiliary tract adenocarcinomas can have squamous components. For any adenocarcinoma in hepatic aspirates, CC, HCC with pseudoacini and CHCC-CC have to be considered.

• **Squamous cell carcinoma: **Most are metastatic or arise in the pancreaticobiliary tract. Large, spindly, "tadpole-shaped" or bizarre cells with dense cytoplasm, keratinization and much necrosis may be seen. Adenosquamous variants are not uncommon.

• **Small/intermediate round cell malignancy: **This includes neuroendocrine tumors (NET), small cell undifferentiated carcinomas (SCUC), undifferentiated nasopharnygeal carcinomas and lymphomas [40,49]. Other possibilities include melanoma, Merkel cell tumor, metastatic adenocarcinomas from prostate, stomach and breast (lobular carcinoma), CC, certain sarcomas and HCC, small cell type. Most **NET **are from the gastrointestinal tract (GIT), pancreaticobiliary tract or lung; primary hepatic NET is unusual [50].**SCUC **usually originates in the lung. Hepatic metastases from **nasopharnygeal carcinomas **tend to be markedly necrotic mimicking abscesses radiologically. **Lymphoma **seldom presents as a primary neoplasm although hepatic involvement is common in advanced disease [51]. It has been reported in hepatitis B virus infection, systemic lupus erythematosus, primary biliary cirrhosis and acquired immunodeficiency syndrome. A high index of suspicion is necessary. It can be mistaken for poorly differentiated carcinoma or HCC. Inflammatory processes, such as inflammatory pseudotumors [52-54], and sinusoidal hematopoietic cells have first to be excluded. Nodular extramedullary hematopoiesis can rarely mimic metastases.

• **Clear cell malignancy: **This can also arise in the kidney, adrenal and ovary [55]. Renal cell carcinoma contains transgressing endothelium and papillary endothelium in fibrovascular cores but lacks peripheral endothelium. Nuclear features also differ. However, renal cell carcinoma has not been called the great mimic for no good reason. Metastases can occur years later. Other pitfalls include hepatocytes with fatty change and metastases from liposarcoma and signet-ring cell adenocarcinoma.

• **Pleomorphic cell malignancy: **This includes large cell undifferentiated carcinomas (LCUC), large cell lymphomas, germ cell tumors and various sarcomas. LCUC is not a pure or single entity; tumors may show glandular and neuroendocrine differentiation. Common sites are lung, GIT and female genital tract.

• **Spindle cell malignancy: **Well-differentiated spindle cell tumors include leiomyosarcoma (LS), neurogenic tumors and fibroblastic/stromal tumors, including gastrointestinal stromal tumor (GIST) [56]. At the poorly differentiated end, LS, malignant fibrous histiocytoma, undifferentiated sarcoma or even sarcomatoid HCC or CC with a spindle cell component, have to be considered. Apart from distinguishing **LS **from leiomyoma, other differential diagnoses include benign reactive processes [57]. The epithelioid variant of LS occurs primarily in the GIT. Hepatic metastases can be mistaken for epithelial tumors, such as, HCC, CC, metastatic carcinoma and melanoma. **GIST **also has spindle and epithelioid cell types [58]. Metastatic GIST may pose diagnostic problems due to their variable morphologic spectrum and cytologic atypia; c-kit staining is necessary. Hepatic angiosarcoma associated with Thorotrast and epithelioid hemangioendothelioma are rare. In the young, differential diagnoses of spindle cell lesions include inflammatory pseudotumor, infantile hemangioendothelioma, mesenchymal hamartoma and undifferentiated (embryonal) sarcoma.

• **Giant cell malignancy: **This can be carcinomatous or sarcomatous. Giant cell carcinomas have been noted in lung, pancreas, thyroid, kidney and breast. Hepatic metastases have to be distinguished from HCC, giant cell type.

• **Hepatoid carcinoma; AFP-producing carcinoma: **Primary hepatic tumors are not the only source of AFP. Neither is the liver the only site of origin for hepatocytic-looking carcinomas. Most of them arise in the lung and GIT. Those with **hepatoid **features mimic classic HCC in having a proclivity for vascular permeation and distant metastases, in this case to the liver. Others produce AFP but are **non-hepatoid**; being usually adenocarcinoma, undifferentiated carcinoma and small or large cell neuroendocrine carcinoma [59].

Suffice it to say that when dealing with FNAB of liver masses, one must be fully aware of any past history of malignancy or rule out any hitherto undetected cancer. Some of the limitations in the categorization of tumors obtained by FNAB can be overcome by immunohistochemistry. The advantages of an exact cytodiagnosis are obvious – it may save the patient a diagnostic laparotomy, especially in inoperable cases, and allow for specific chemotherapy to be instituted without delay. However, at best, information gleaned from a precise cytodiagnosis can sometimes only favor a particular primary site.

### Step 4: Further confirm nature of cytohistologic findings

The initial cytologic assessment is crucial as it forms the basis upon which ancillary tests are ordered; the results of which should be interpreted in the larger context of the case.

#### Special stains

• **Reticulin**

Study of the reticulin framework, stained by Gomori's method, is important in the analysis of well-differentiated hepatocellular nodules [60,61]. HCC have deficient or absent reticulin; the reticulin framework in LCA and FNH may not be well developed either.

• **Periodic acid-Schiff with and without diastase; Mucicarmine**

This is usually performed to distinguish glycogen from epithelial mucin. Hepatocytes are loaded with glycogen. Cells exhibiting glandular (biliary) differentiation may show intracytoplasmic or intraluminal mucin production.

• **Fat**

Fat can be demonstrated in nonneoplastic and neoplastic hepatocytes by staining fresh or formalin-fixed tissue with Oil Red O. It has no discriminant value in defining the biological status of hepatocytes but may be of help in deciphering the contents of cytoplasmic vacuoles of cells of unrecognizable histogenesis.

• **Iron**

Iron appears as black cytoplasmic granules in hepatocytes stained with Giemsa stains and dark brown with Papanicolaou stains. The pigment can be confirmed with Perl's prussian blue method. Iron accumulation in hepatocytes favors a benign process. On the other hand, in populations where hepatic iron accumulation is common, the absence of iron in hepatocytes should alert one to the presence of a proliferative process, be it regenerative or neoplastic.

#### Immunohistochemistry

A whole battery of antibodies is available for the comparative immunohistochemical study of primary and metastatic liver tumors. The two major diagnostic issues are (i) whether the hepatocytes are malignant or benign, and (ii) what the histogenesis of the malignant cells is [See *"Diagnostic utility of immunohistochemistry"*].

### Step 5: Establish final diagnosis based on multidisciplinary approach

Close clinicopathologic correlation is mandatory for enhancing the yield of FNAB diagnoses and the reduction of indeterminate reports.

## Current diagnostic issues

### Reappraisal of role of hepatic fine needle aspiration biopsy

#### 1. It is still to procure a tissue diagnosis as part of the evaluation of focal liver lesions but under circumstances where the clinical, biochemical and imaging profiles are not conclusive

A benign cytodiagnosis obviates unnecessary surgery. Surgical resection is indicated for any resectable malignant hepatic mass, be it primary or secondary. In unresectable malignant lesions, a precise cytohistologic typing is crucial for appropriate alternative therapy. The need for biopsy diagnosis in HCC is now a hotly debated topic [2,4,12]. The stand in some practices is that a needle biopsy may be indicated only if it is not possible to diagnose HCC by other means, namely, serum AFP concentration, spiral computed tomography (CT) and magnetic resonance imaging (MRI). In advanced HCC, the need for biopsy may be obviated due to the non-availability of effective therapy. In early/small HCC, even if a biopsy is performed, the diagnostic sensitivity of the procedure may be as low as 60% [3]. If there is a high index of suspicion for HCC despite negative cytologic sampling, specific therapy may be instituted.

#### 2. It is a safe and well-tolerated, minimally invasive procedure with low risk of complications in suitable candidates and in skilled hands

Needle track seeding by malignant cells is the main reason often cited by opponents of FNAB of the liver [2,62-64]. There is no reliable data to establish the risk; the figure of 0.006% is regarded as a gross underestimation by many authors [3,65,66]. Needle biopsies, in general, are usually not indicated in patients deemed suitable for liver transplantation due to possible seeding [5]. The move to institute definitive treatment for classic HCC diagnosed solely on clinical, biochemical and radiologic grounds without tissue confirmation has crept slowly into some practices. The risk of false positive diagnosis of HCC with subsequent aggressive therapy, such as liver transplantation, by far outweighs the risk of seeding.

#### 3. It is a technique with high sensitivity and specificity when practised in a multidisciplinary setting by skilled operators

Tissue procurement by FNAB under radiologic guidance and cytologic interpretation of the aspirated material are both highly operator-dependent. An experienced screener on-site can give a rapid assessment of adequacy.

#### 4. Surveillance for early HCC in high-risk patients has resulted in the detection of "suspicious" nodules in cirrhotic livers that are often <2 cm in diameter

The usual surveillance tools are serum AFP concentration and ultrasonography (US). AFP is not a very good screening test since it has a sensitivity of 39–64%, a specificity of 76–91% and a positive predictive value of between 9–32% [6,67]. On the other hand, US has, as a screening test in hepatitis B surface antigen (HBsAg) carriers, a sensitivity of 71% and specificity of 93%, but its positive predictive value is only 14% [6]. Although a spectrum of hepatocellular nodular lesions can be encountered, especially in a background of cirrhosis, studies have revealed that about half of them are not HCC. Tissue characterization is, therefore, mandatory and FNAB may be the simplest and most practical means to reach small, deep-seated lesions [1].

*The ****European Association for the Study of the Liver ****(Barcelona-2000 EASL Conference) *[5]*has drawn up guidelines for the current clinical management of HCC. HCC can be diagnosed in a setting of liver cirrhosis if a focal liver lesion is >2 cm in diameter with arterial hypervascularization, and shows coincident features in at least two imaging techniques (US, spiral CT, MRI and angiography); or characteristic features in one imaging technique associated with serum AFP level of >400 ng/ml. It follows then that there is no necessity to obtain cytologic and/or histologic confirmation for all cases of suspected HCC. It is recommended that punctures be limited to patients with nodules <2 cm diameter for the differentiation of HCC from regenerative nodules. In the future, tumor biopsy may assume a different role by becoming a useful tool for procuring tissue for the molecular profiling of the disease. It is important to note that the above recommendations are not meant for everyone. It should be carried out in highly selected patients by highly skilled healthcare teams in specialized tertiary centers.*

### Fine needle aspiration biopsy versus core needle biopsy

**Fine needle aspiration biopsy **is useful for (i) cirrhotic patients with poor liver function with risk of bleeding; (ii) liver masses with obstructive jaundice and risk of bile leakage, those near big vessels, or where there is need to go through bowel; (iii) small (<2 cm diameter), deep-seated and difficult to approach nodules that require close patient co-operation during the procedure; (iv) representative sampling of sizeable lesions by re-direction of the needle and multiple passes; and (v) on-site rapid assessment of adequacy and rendering of provisional diagnosis, as well as for appropriate triage of tissue specimens for ancillary studies (e.g. microbiology, flow cytometry, genetic testing, molecular diagnostics, cell block preparation and electron microscopy) [22].

**Core needle biopsy **(CNB), with the availability of more material, provides tissue for histologic and immunohistochemical studies, especially in two major areas of diagnostic difficulties, namely, in the (i) differentiation of WD-HCC from benign hepatocellular nodules; and (ii) separation of HCC from CC and metastases. A critical comparative evaluation of the risks of seeding by CNB and FNAB is needed. For the proponents of CNB, the view is that a single pass with larger bore needles (<20 gauge) may be preferable to multiple passes by finer needles needed to obtain sufficient material for cytohistologic examination. The incidence of seeding may increase as a result of the number of punctures of tumors detected at an early stage in patients with a longer life expectancy [66].

**Consensus: **The diagnostic accuracy in terms of sensitivity, specificity and positive predictive value of FNAB for HCC is almost similar to that of CNB. The accuracy rate is highly operator-dependent and increases with both techniques combined. The specificity and positive predictive value of FNAB in the diagnosis of malignant hepatic lesions has been shown to be close to 100% in most studies [19,22,68,69]. These results are comparable to the accuracy of CNB. In a comparative study, it was reported that both procedures, FNAB and CNB, had the same diagnostic accuracy of 78% when considered separately and of 88% when considered in combination [21]. The conclusion was that the great advantage of combining the two techniques was the reduction in false negative results. Performing both procedures at the same sitting may not be feasible due to medical contraindications and may also not be acceptable clinical practice. Using larger caliber cutting needle biopsies can be associated with a greater number of complications [68]. This quagmire can be overcome by using cutting FNAB needles. At our institution, the utilization of such (21 gauge) aspiration needles has provided us with ample material for smear preparations as well as tissue cores resembling microbiopsies rather than mere cell blocks [20,34]. Our practice has been to retrieve sizeable particulate matter from the material on the glass slides prior to smearing and fixing them in formalin for cell block preparation. Many studies have attested to the improved diagnostic yield and accuracy of FNAB using the combined cytohistologic approach [23,70]

FNAB can provide rapid on-site diagnosis when the smears are stained with Diff-Quik or Ultra-fast Papanicolaou stain [28]. In the era of rising costs in medical practice and higher patient/practitioner/institution expectations of efficiency and faster turn-around time, FNAB can obviate the need to wait for tissue processing if accurate cytologic diagnoses can be rendered. Another cost-saving advantage, especially for less developed countries, is that smears are cheap, convenient and easy to prepare as long as there is an experienced person to interpret them.

Considering the overall advantages and cost-analysis, FNAB can be suggested as the initial method of choice for evaluation of focal liver lesions in most clinical settings. The final choice should be decided on the basis of the working clinical diagnosis and the institutional/personal experience.

### Separation of well-differentiated hepatocellular carcinoma from benign hepatocellular nodular lesions

#### Factors that pose diagnostic problems and pitfalls

1. HCC can be small and focal, solitary and large, multifocal, or diffuse and infiltrating; thereby, mimicking small benign lesions on the one hand and metastases on the other, especially in imaging studies.

2. Serum AFP, though fairly specific, has poor sensitivity for the diagnosis of HCC, regardless of tumor size or degree of differentiation. There is significant elevation in about 50–60% of HCC [24,33]. Small WD-HCC are usually not associated with serum AFP elevation. On the other hand, transient increases may be seen with inflammatory flares in chronic viral hepatitis. Published data at the current moment suggest using values of >400 ng/ml for diagnostic confirmation of HCC [67].

3. The diagnostic dilemma at the highly WD-HCC end is that the hepatocytic histogenesis is obvious but proof of malignancy may be lacking [24,32,33]. The cell cords tend not to be >2 cells thick and the cellular pleomorphism and subtle increase in the N/C ratios of the hepatocytes may not be appreciated under light microscopy. In fact, highly WD-HCC tend to be composed of small, uniform, strikingly monotonous neoplastic hepatocytes with slightly increased N/C ratios, imparting an impression of nuclear crowding [34]. The recognition of polymorphism with variation in cell and nuclear sizes and a normal N/C ratio of 1/3 should alert one to the likelihood of benignity of the hepatocytes [20].

4. Routine surveillance by more than one imaging technique in high-risk patients tends to detect nodule/s of various natures in the cirrhotic livers [10,71]. HCC tend to occur in a cirrhotic background together with MRN and low- and high-grade DN. Pure light microscopic interpretation with immunohistochemical input may no longer suffice in the diagnostic workup towards an accurate tissue diagnosis.

5. Smaller and smaller lesions (<2 cm diameter) are being increasingly detected by imaging and subjected to FNAB for tissue diagnosis.

6. Early HCC tend to be small and highly well-differentiated, making differentiation from MRN and DN difficult. Increasing numbers of equivocal nodular lesions are being detected in cirrhotic livers by various imaging modalities. The histopathologic interpretation of these nodules is highly controversial, let alone cytologic assessment. Eastern pathologists tend to call them early HCC while the Western fraternity tends to go for a diagnosis of high-grade DN [14,17].

7. Small HCC are prone to fatty change due to inadequate neoangiogenesis; poor vascularity makes them difficult to visualize and characterize by imaging methods [8,14]. Increasing degree of heterogeneity is exhibited as the lesion enlarges [15].

8. Well-differentiated hepatocellular nodules of any nature can occur in non-cirrhotic livers and have to be distinguished from each other and from the surrounding liver.

9. Fatty change can occur in WD-HCC, LCA, FNH and DN without associated steatosis in the surrounding liver parenchyma. An entity called focal fatty alteration/change can also mimic these nodules [20,36].

10. Current histologic criteria for the diagnosis of highly WD-HCC include loss of reticulin, thickening of trabeculae (>2 cells thick), sinusoidal capillarization, unpaired arterioles, cellular pleomorphism/heterogeneity, "clonal" growth patterns, pseudoacini, mitotic activity [14,16] and stromal invasion of portal tracts. Some of these criteria are lacking in cytologic material.

11. In high-risk cases, some "non-malignant" nodules with large cell change (low-grade DN) or small cell change (high-grade DN) may be precursor lesions [11,18,72-74]. Morphometric studies suggest that small cell change may be the more sinister lesion biologically. HCC occurs in 5–40% of cirrhotic patients and foci of cancer are found in a third of DN [75]. Some of these precursor lesions may be indistinguishable from malignant hepatocellular nodules by light microscopy alone.

12. Current concepts on how premalignant lesions develop and how HCC may arise within them impact on the accuracy of pathologic diagnosis of hepatocellular nodules [7,9,10]. Inadequacies of FNAB evaluation of hepatocellular nodules cannot be ignored given the focality of proliferative clones of atypical cells within the nodules and the shortcomings of pure morphologic interpretation.

### Diagnostic utility of immunohistochemistry

There are two major applications for immunohistochemical markers in the diagnostic workup of focal liver lesions [20,76]. One is to decipher the exact histogenetic origin of obvious tumor nodules – that is, the histologic typing and the primary site [77]. It may not always be possible to distinguish between the poorly differentiated entities of HCC, CC and metastatic carcinomas [25,78]. By the same token, adenocarcinomas occurring in the liver may be metastatic or primary in origin. Of interest lately is the increasing documentation of AFP-producing extrahepatic hepatoid/non-hepatoid carcinomas that have a propensity for vascular invasion and liver metastases [59,79]. The immunoprofile of these tumors, originating mostly in the GIT and lungs, is almost identical to that of HCC. Serum AFP levels tend to be very high. The other application concerns the distinction of the various lesions within the realm of well-differentiated hepatocellular nodular lesions. [See "*FNAB of well-differentiated hepatocellular nodular lesions*"]. For ascertainment of malignancy in hepatocellular nodules, the antibody panel should comprise at least AFP, *p*CEA or CD10, and CD34 [80-82]. Additional markers, such as Hep Par 1 and cytokeratins, should be included if the histogenesis of the tumor is to be studied. Markers of cell proliferation, proliferating cell nuclear antigen (PCNA) and Ki67; and p53 are not routinely used.

**Figure 11:**
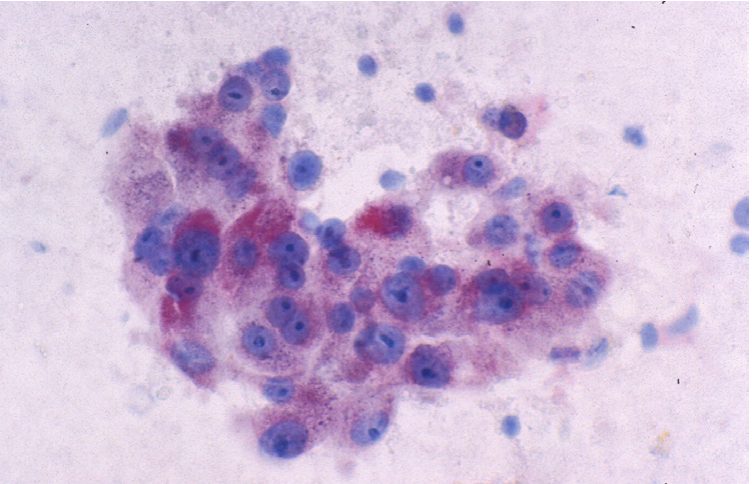
***Hepatocellular carcinoma: FNAB***. The tumor cells stain positively for alpha-fetoprotein (Immunostain).

**Figure 12:**
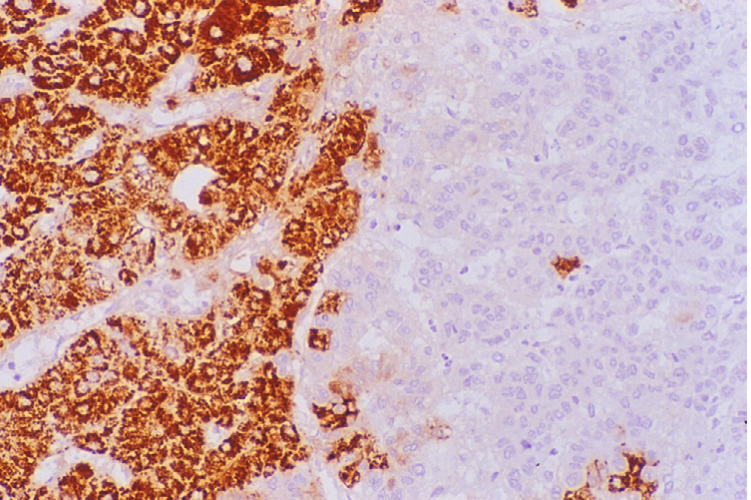
***Hepatocellular carcinoma: Histology***. Parts of the tumor show intense granular cytoplasmic reactivity with HepPar1 (Immunostain).

• **AFP **is fairly specific but not sensitive for HCC. Tissue AFP immunoreactivity is expected in HCC (Figure 11) but it may be patchy and minimal. Sensitivity is about 50% (range, 20–75%) and is low at both ends of the histologic spectrum of HCC [44,83-86]. A study of 56 patients with small HCC (<2 cm diameter) showed AFP-positivity in 44.6% of the tumors [87]. A variable staining pattern may be encountered with CHCC-CC. MRN in cirrhosis are not associated with elevated serum or stainable tissue AFP [88]; neither are DN or LCA. The specificity of AFP for HCC is being challenged by reports of AFP-producing extrahepatic hepatoid/non-hepatoid carcinomas [59]. There is an increasing tendency to drop this immunomarker from the panel for HCC workup.

• ***p*CEA **stains bile canaliculi and ductal epithelium but not hepatocytes. A characteristic "chicken-wire fence" canalicular staining pattern is seen in normal liver [89]. Biliary epithelial cells show diffuse cytoplasmic and brush border staining. A normal canalicular pattern is expected for benign hepatocellular nodular lesions. However, some LCA and even, some FNH, may exhibit deficient canaliculi. There are two patterns of staining in HCC – canalicular and/or diffuse cytoplasmic staining. The canalicular pattern is abnormal and deficient in HCC with twisted and often dilated structures; it may not even be appreciable in high-grade HCC [78]. Instead, diffuse cytoplasmic staining may be seen in less differentiated HCC, making distinction from a poorly differentiated adenocarcinoma difficult. In the context of a carcinoma, a canalicular pattern is specific for HCC.

• **CD10 **is expressed in normal and neoplastic liver, exhibiting a similar canalicular staining pattern to *p*CEA [90,91]. Although it does not differentiate between benign and malignant hepatocellular nodular lesions, CD10 is very useful in distinguishing HCC from non-HCC malignancies. The sensitivity of CD10 (68.3%) is far better than immuno-staining for AFP (23.8%) but less sensitive than *p*CEA (95.2%) in the diagnosis of HCC [92].

• **Hep Par 1 (Hepatocyte antigen) **is a sensitive marker for hepatocytic differentiation and is part of the antibody panel for distinguishing HCC from CC and metastases. However, not all HCC stain uniformly (Figure 12) and not all Hep Par 1-positive tumors are of hepatocellular origin or arise in the liver [86,93-96]. Its variable and heterogeneous staining pattern, which can range from 100% -positive cells in WD-HCC to <5% in some PD-HCC cases, may lead to false negative results in small samples [97]. MRN, DN, FNH and LCA tend to exhibit 100% positivity. Hence, this antibody has no discriminant value in the evaluation of the biological status of well-differentiated hepatocellular nodular lesions.

• **Cytokeratins **(CK 7, 8, 18, 19, 20; CAM 5.2; AE1/AE3). Mature hepatocytes stain with CK 8 and 18 and CAM 5.2 but not with CK 7, 19 or 20 or AE1/AE3. CAM 5.2 is the most reliable cytokeratin antibody for HCC. AE1/AE3 negativity is expected in hepatocellular lesions. Focal CK 7 and 19 positivity can be seen in high-grade HCC. HCC is generally CK 20 negative [98].

• **CD34 **is an intercellular adhesion protein found in normal endothelium but absent in normal sinusoids. CD34 highlights regions of sinusoidal capillarization where there is basement membrane material deposition. Diffuse sinusoidal CD34 reactivity is seen in HCC, even small WD-HCC [99]. However, significant reactivity is also seen in LCA and some FNH. In cirrhotic and DN, staining is absent or minimal, and confined to the periportal/periseptal regions [100].

## Conclusion

Tissue confirmation is recommended in the diagnosis of focal liver lesions as the risk of aggressive therapy is greater than the risk of malignant seeding. FNAB has many advantages that CNB lacks. The lack of tissue can be overcome by using cutting aspiration needles that can provide material for smear cytology and microhistology. Guided FNAB is a highly operator-dependent procedure as is the preparation and interpretation of the cytologic material. Optimization of FNAB in the diagnosis of focal liver lesions, increase in the yield of true positive diagnoses, and rendering of fewer indeterminate reports require close clinicopathologic correlation; combination of smear cytology and microhistology, use of a discriminant panel of special and immunostains; and team work by skilled operators on all fronts.

## List of abbreviations

FNAB : Fine needle aspiration biopsy

HCC : Hepatocellular carcinoma

MRN : Macroregenerative nodule

DN : Dysplastic nodule

FNH : Focal nodular hyperplasia

LCA : Liver cell adenoma

WD-HCC : Well-differentiated hepatocellular carcinoma

PD-HCC : Poorly differentiated hepatocellular carcinoma

CC : Cholangiocarcinoma

AFP : Alpha-fetoprotein

CEA : Carcinoembyronic antigen

N/C ratio : nuclear-cytoplasmic ratio

CHCC-CC : Combined hepatocellular-cholangiocarcinoma

*p*CEA : polyclonal carcinoembyronic antigen

NET : Neuroendocrine tumor

SCUC : Small cell undifferentiated carcinoma

GIT : Gastrointestinal tract

LCUC : Large cell undifferentiated carcinoma

LS : Leiomyosarcoma

GIST : Gastrointestinal stromal tumor

CT : Computed tomography

MRI : Magnetic resonance imaging

US : Ultrasonography

CNB : Core needle biopsy

PCNA : Proliferating cell nuclear antigen

## Competing interests

The author(s) declare that they have no competing interests.

## Authors' contributions

This is solely my work.

## References

[B1] Caturelli E, Ghittoni G, Roselli P, De Palo M, Anti M (2004). Fine needle biopsy of focal liver lesions: The hepatologist's point of view. Liver Transpl.

[B2] Cetta F, Zuckermann M, De Nisi A (2001). Is needle biopsy of the liver necessary to diagnose HCC?. Gut.

[B3] Durand F, Regimbeau JM, Belghiti J, Sauvanet A, Vilgrain V, Terris B, Moutardier V, Farges O, Valla D (2001). Assessment of the benefits and risks of percutaneous biopsy before surgical resection of hepatocellular carcinoma. J Hepatol.

[B4] Llatjos M, Muns R, Tallada N (2001). Need for biopsy in hepatocellular carcinoma. J Hepatol.

[B5] Bruix J, Sherman M, Llovet JM, Beaugrand M, Lencioni R, Burroughs AK, Christensen E, Pagliaro L, Colombo M, Rodes J (2001). Clinical management of hepatocellular carcinoma. Conclusions of the Barcelona – 2000 EASL Conference. J Hepatol.

[B6] Collier J, Sherman M (1998). Screening for hepatocellular carcinoma. Hepatology.

[B7] Borzio M, Borzio F, Croce A, Sala M, Salmi A, Leandro G, Bruno S, Roncalli M (1997). Ultrasonography-detected macroregenerative nodules in cirrhosis: a prospective study. Gastroenterology.

[B8] Kutami R, Nakashima Y, Nakashima O, Shiota K, Kojiro M (2000). Pathomorphologic study on the mechanism of fatty change in small hepatocellular carcinoma of humans. J Hepatol.

[B9] Terasaki S, Kaneko S, Kobayashi K, Nonomura A, Nakanuma Y (1998). Histological features predicting malignant transformation of nonmalignant hepatocellular nodules: a prospective study. Gastroenterology.

[B10] Theise ND (1995). Macroregenerative (dysplastic) nodules and hepatocarcinogenesis: Theoretical and clinical considerations. Semin Liver Dis.

[B11] International Working Party (1995). Terminology of nodular hepatocellular lesions. Hepatology.

[B12] Torzilli G, Minagawa M, Takayama T, Inoue K, Hui AM, Kubota K, Ohtomo K, Nakuuchi M (1999). Accurate preoperative evaluation of liver mass lesions without fine-needle biopsy. Hepatology.

[B13] Kimura H, Nakajima T, Kagawa K, Deguchi T, Kakusui M, Katagishi T, Okanoue T, Kashima K, Ashihara T (1998). Angiogenesis in hepatocellular carcinoma as evaluated by CD 34 immunohistochemistry. Liver.

[B14] Kojiro M (2004). Focus on dysplastic nodules and early hepatocellular carcinoma: An Eastern point of view. Liver Transpl.

[B15] Nakashima Y, Nakashima O, Hsia CC, Kojiro M, Tabor E (1999). Vascularization of small hepatocellular carcinomas: Correlation with differentiation. Liver.

[B16] Quaglia A, Bhattacharjya S, Dhillon AP (2001). Limitations of the histopathological diagnosis and prognostic assessment of hepatocellular carcinoma. Histopathology.

[B17] Roncalli M (2004). Hepatocellular nodules in cirrhosis: Focus on diagnostic criteria on liver biopsy. A Western experience. Liver Transpl.

[B18] Ishak KG, Goodman ZD, Stocker JT (2001). Tumors of the liver and intrahepatic bile ducts. Atlas of Tumor Pathology 3rd series Fascicle 31.

[B19] Hertz G, Reddy VB, Green L, Spitz D, Massarani-Wafai R, Selvaggi SM, Kluskens L, Gattuso P (2000). Fine-needle aspiration biopsy of the liver: A multicenter study of 602 radiologically guided FNA. Diagn Cytopathol.

[B20] Wee A, Sampatanukul P (2004). Fine needle aspiration cytology of the liver. Diagnostic algorithms. A Southeast Asian perspective.

[B21] Franca AVC, Valerio HMG, Trevisan M, Escanhoela C, Seva-Pereira T, Zucoloto S, Martinelli A, Soares EC (2003). Fine needle aspiration biopsy for improving the diagnostic accuracy of cut needle biopsy of focal liver lesions. Acta Cytol.

[B22] Jain D (2002). Diagnosis of hepatocellular carcinoma. Fine needle aspiration cytology or needle core biopsy. J Clin Gastroenterol.

[B23] Longchampt E, Patriache C, Fabre M (2000). Accuracy of cytology vs. microbiopsy for the diagnosis of well-differentiated hepatocellular carcinoma and macroregenerative nodule. Definition of standardized criteria from a study of 100 cases. Acta Cytol.

[B24] Wee A, Nilsson B, Tan LKA, Yap I (1994). Fine needle aspiration biopsy of hepatocellular carcinoma: Diagnostic dilemma at the ends of the spectrum. Acta Cytol.

[B25] Das DK (1999). Cytodiagnosis of hepatocellular carcinoma in fine-needle aspirates of the liver: Its differentiation from reactive hepatocytes and metastatic adenocarcinoma. Diagn Cytopathol.

[B26] Ferrell L (1995). Malignant liver tumors that mimic benign lesions: Analysis of five distinct lesions. Semin Diagn Pathol.

[B27] Pisharodi LR, Lavoie R, Bedrossian CWM (1995). Differential diagnostic dilemmas in malignant fine-needle aspirates of liver: A practical approach to final diagnosis. Diagn Cytopathol.

[B28] Yang GC, Yang GY, Tao LC (2004). Cytologic features and histologic correlations of microacinar and microtrabecular types of well-differentiated hepatocellular carcinoma in fine-needle aspiration biopsy. Cancer (Cancer Cytopathol).

[B29] Sole M, Calvet X, Cuberes T, Maderuelo F, Bruix J, Bru C, Rey MJ, Serna N, Cardesa A (1993). Value and limitations of cytologic criteria for the diagnosis of hepatocellular carcinoma by fine needle aspiration biopsy. Acta Cytol.

[B30] Soyuer I, Ekinci C, Kaya M, Genc Y, Bahar K (2003). Diagnosis of hepatocellular carcinoma by fine needle aspiration cytology: Cellular features. Acta Cytol.

[B31] Wee A, Nilsson B, Chan-Wilde C, Yap I (1991). Fine needle aspiration biopsy of hepatocellular carcinoma: Some unusual features. Acta Cytol.

[B32] de Boer WB, Segal A, Frost FA, Sterrett GF (1999). Cytodiagnosis of well differentiated hepatocellular carcinoma: Can indeterminate diagnoses be reduced?. Cancer.

[B33] Wee A, Nilsson B (2003). Highly well differentiated hepatocellular carcinoma and benign hepatocellular lesions: Can they be distinguished on fine needle aspiration biopsy?. Acta Cytol.

[B34] Takenaka A, Kaji I, Kasugai H, Sasaki Y, Ishiguro S, Wada A, Horai T, Otani T, Ishikawa H (1999). Usefulness of diagnostic criteria for aspiration cytology of hepatocellular carcinoma. Acta Cytol.

[B35] Layfield LJ (1994). Focal fatty change of the liver: Cytologic findings in a radiographic mimic of metastases. Diagn Cytopathol.

[B36] Zeppa P, Anniciello A, Vetrani A, Palombini L (2002). Fine needle aspiration biopsy of hepatic focal fatty change: A report of two cases. Acta Cytol.

[B37] Gupta SK, Das DK, Rajwanshi A, Bhusnurmath SR (1986). Cytology of hepatocellular carcinoma. Diagn Cytopathol.

[B38] Singh HK, Silverman JF, Geisinger KR (1997). Fine-needle aspiration cytomorphology of clear-cell hepatocellular carcinoma. Diagn Cytopathol.

[B39] Piatti B, Caspani B, Giudici C, Ferrario D (1997). Fine needle aspiration biopsy of hepatocellular carcinoma resembling neuroendocrine tumor: A case report. Acta Cytol.

[B40] Wee A, Nilsson B, Yap I (1996). Fine needle aspiration biopsy of small/intermediate cell tumors in the liver: Considerations in a Southeast Asian population. Acta Cytol.

[B41] Maeda T, Adachi E, Kajiyama K, Takenaka K, Sugimachi K, Tsuneyoshi M (1996). Spindle cell hepatocellular carcinoma: A clinicopathologic and immunohistochemical analysis of 15 cases. Cancer.

[B42] Davenport RD (1990). Cytologic diagnosis of fibrolamellar carcinoma of the liver by fine needle aspiration. Diagn Cytopathol.

[B43] Perez-Guillermo M, Masgrau NA, Garcia-Solano J, Sola-Perez J, de Agustin y de Agustin P (1999). Cytologic aspect of fibrolamellar hepatocellular carcinoma in fine-needle aspirates. Diagn Cytopathol.

[B44] Wu PC, Fang JWS, Lau VKT, Lai CL, Lo CK, Lau JYN (1996). Classification of hepatocellular carcinoma according to hepatocellular and biliary differentiation markers: Clinical and biological implications. Am J Pathol.

[B45] Vessey CJ, de la Hall PM (2001). Hepatic stem cells: A review. Pathology.

[B46] Jarnagin WR, Weber S, Tickoo SK, Koea JB, Obiekwe S, Fong Y, DeMatteo RP, Blumgart LH, Klimstra D (2002). Combined hepatocellular and cholangiocarcinoma: demographic, clinical, and prognostic factors. Cancer.

[B47] Wee A, Nilsson B (1999). Combined hepatocellular-cholangiocarcinoma: Diagnostic challenge in hepatic fine needle aspiration biopsy. Acta Cytol.

[B48] Tsui WM, Lam PW, Mak CK, Pay KH (2000). Fine-needle aspiration cytologic diagnosis of intrahepatic biliary papillomatosis (intraductal papillary tumor): Report of three cases and comparative study with cholangiocarcinoma. Diagn Cytopathol.

[B49] Pisharodi LR, Bedrossian C (1998). Diagnosis and differential diagnosis of small-cell lesions of the liver. Diagn Cytopathol.

[B50] Prosser JM, Dusenbery D (1997). Histocytologic diagnosis of neuroendocrine tumors in the liver: A retrospective study of 23 cases. Diagn Cytopathol.

[B51] Collins KA, Geisinger KR, Raab SS, Silverman JF (1996). Fine needle aspiration biopsy of hepatic lymphomas: Cytomorphology and ancillary studies. Acta Cytol.

[B52] Malhotra V, Gondal R, Tatke M, Sarin SK (1997). Fine needle aspiration cytologic appearance of inflammatory pseudotumor of the liver: A case report. Acta Cytol.

[B53] Nakama T, Hayashi K, Komada N, Ochiai T, Hori T, Shiori S, Tsubouchi H (2000). Inflammatory pseudotumor of the liver diagnosed by needle liver biopsy under ultrasonographic tomography guidance. J Gastroenterol.

[B54] Wee A, Nilsson B, Yap I, Chong SM (1995). Aspiration cytology of liver abscesses: With an emphasis on diagnostic pitfalls. Acta Cytol.

[B55] Hughes JH, Jensen CS, Donnelly AD, Cohen MB, Silverman JF, Geisinger KR, Raab SS (1999). The role of fine-needle aspiration cytology in the evaluation of metastatic clear cell tumors. Cancer.

[B56] Powers CN, Berardo MD, Frable WJ (1994). Fine-needle aspiration biopsy: Pitfalls in the diagnosis of spindle-cell lesions. Diagn Cytopathol.

[B57] Tao LC, Davidson DD (1993). Aspiration biopsy cytology of smooth muscle tumors: A cytologic approach to the differentiation between leiomyosarcoma and leiomyoma. Acta Cytol.

[B58] Padilla C, Saez A, Catala I, Vidal A, Garcia L, Tolosa F, Javier Andreu F, Combalia N (2002). Fine-needle aspiration cytology diagnosis of metastatic gastrointestinal stromal tumor in the liver. Diagn Cytopathol.

[B59] Wee A, Thamboo TP, Thomas A (2003). Alpha-fetoprotein-producing liver carcinomas of primary extrahepatic origin: Fine needle aspiration biopsy experience in 2 cases. Acta Cytol.

[B60] Bergman S, Graeme-Cook F, Pitman MB (1997). The usefulness of the reticulin stain in the differential diagnosis of liver nodules on fine-needle aspiration biopsy cell block preparations. Mod Pathol.

[B61] Gagliano EF (1995). Reticulin stain in the fine needle aspiration differential diagnosis of liver nodules. Acta Cytol.

[B62] Cedrone A, Rapaccini GL, Pompili M, Grattagliano A, Aliotta A, Trombino C (1992). Neoplastic seeding complicating percutaneous ethanol injection for treatment of hepatocellular carcinoma. Radiology.

[B63] Smith EH (1991). Complications of percutaneous abdominal fine-needle biopsy. Radiology.

[B64] Takamori R, Wong LL, Dang C, Wong L (2000). Needle-tract implantation from hepatocellular carcinoma: is needle biopsy of the liver always necessary?. Liver Transpl.

[B65] Huang GT, Sheu JC, Yang PM, Lee HS, Wang TH, Chen DS (1996). Ultrasound-guided cutting biopsy for the diagnosis of hepatocellular carcinoma – a study based on 420 patients. J Hepatol.

[B66] Schotman SN, De Man RA, Stoker J, Zondervan PE, IJzermans JNM (1999). Subcutaneous seeding of hepatocellular carcinoma after percutaneous needle biopsy. Gut.

[B67] Sherman M (2001). Alphafetoprotein: an orbituary. J Hepatol.

[B68] Buscarini L, Fornari F, Bolondi L, Colombo P, Livraghi T, Magnolfi F, Rapaccini GL, Salmi A (1990). Ultrasound-guided fine-needle biopsy of focal liver lesions: techniques, diagnostic accuracy and complications. A retrospective study on 2091 biopsies. J Hepatol.

[B69] Fornari F, Civardi G, Cavanna L, Rossi S, Buscarini E, Di Stasi M, Sbolli G, Buscarini L (1990). Ultrasonically guided fine-needle aspiration biopsy: a highly diagnostic procedure for hepatic tumors. Am J Gastroenterol.

[B70] Caturelli E, Bisceglia M, Fusilli S, Squillante MM, Castelvertere M, Siena DA (1996). Cytological vs microhistological diagnosis of hepatocellular carcinoma: Comparative accuracies in the same fine-needle biopsy specimen. Dig Dis Sci.

[B71] Zhou H, Wolff M, Pauliet D, Fischer HP, Pfeifer U (2000). Multiple macroregenerative nodules in liver cirrhosis due to Budd-Chiari syndrome: Case reports and review of the literature. Hepatogastroenterology.

[B72] Ferrell LD, Crawford JM, Dhillon AP, Scheuer PJ, Nakanuma Y (1993). Proposal for standardized criteria for the diagnosis of benign, borderline, and malignant hepatocellular lesions arising in chronic advanced liver disease. Am J Surg Pathol.

[B73] Hytiroglou P, Thiese ND (1998). Differential diagnosis of hepatocellular nodular lesions. Semin Diagn Pathol.

[B74] Wanless IR (1996). Nodular regenerative hyperplasia, dysplasia, and hepatocellular carcinoma. Am J Gastroenterol.

[B75] Terada T, Ueda K, Nakanuma Y (1993). Histopathological and morphometric analysis of atypical adenomatous hyperplasia of human cirrhotic livers. Virchows Arch (A) Pathol Anat Histopathol.

[B76] Goldstein NS, Silverman JF, Dabbs DJ (2002). Immunohistochemistry of the gastrointestinal tract, pancreas, bile ducts, gallbladder, and liver. Diagnostic Immunohistochemistry.

[B77] Gupta RK, Kenwright DN, Naran S, Lallu S, Fauck R (2000). Aspiration cytodiagnosis of small cell malignancies found in fine needle aspirate (FNA) of the liver: An immunocytochemical study. Cytopathology.

[B78] Lau SK, Prakash S, Geller SA, Alsabeh R (2002). Comparative immunohistochemical profile of hepatocellular carcinoma, cholangiocarcinoma, and metastatic adenocarcinoma. Hum Pathol.

[B79] Ishikura H, Kishimoto T, Andachi H, Kakuta Y, Yoshiki T (1997). Gastrointestinal hepatoid adenocarcinoma: venous permeation and mimicry of hepatocellular carcinoma, a report of four cases. Histopathology.

[B80] Saad RS, Luckasevic TM, Noga CM, Fukuda H, Tanikawa K (2004). Diagnostic value of HepPar1, pCEA, CD10, and CD34 expression in separating hepatocellular carcinoma from metastatic carcinoma in fine-needle aspiration cytology. Diagn Cytopathol.

[B81] Stahl J, Voyvodic F (2000). Biopsy diagnosis of malignant versus benign liver "nodules": New helpful markers. An update. Adv Anat Pathol.

[B82] Tsuji M, Kashihara T, Terada N, Mori H (1999). An immunohistochemical study of hepatic atypical adenomatous hyperplasia, hepatocellular carcinoma, and cholangiocarcinoma with alpha-fetoprotein, carcinoembryonic antigen, CA 19-9, epithelial membrane antigen, and cytokeratins 18 and 19. Pathol Int.

[B83] Brumm C, Schulze C, Charels K, Morohoshi T, Kloppel G (1989). The significance of alpha-fetoprotein and other tumor markers in differential immunohistochemistry of primary liver tumors. Histopathology.

[B84] Hurrlimann J, Gardiol D (1991). Immunohistochemical characterization of 130 cases of primary hepatic carcinomas. Am J Surg Pathol.

[B85] Ma CK, Zarbo RJ, Frierson HF, Lee MW (1993). Comparative immunohistochemical study of primary and metastatic carcinomas of the liver. Am J Clin Pathol.

[B86] Minervini MI, Demetris AJ, Lee RG, Carr BI, Madariaga J, Nalesnik MA (1997). Utilization of hepatocyte-specific antibody in the immunocytochemical evaluation of liver tumors. Mod Pathol.

[B87] Sato K, Tanaka M, Kusaba T, Fukuda H, Tanikawa K (1998). Immunohistochemical demonstration of alpha-fetoprotein in small hepatocellular carcinoma. Oncol Rep.

[B88] Theise ND, Fiel IM, Hytiroglou P, Ferrell L, Schwartz M, Miller C, Thung SN (1995). Macroregenerative nodules in cirrhosis are not associated with elevated serum or stainable tissue alpha-fetoprotein. Liver.

[B89] Wee A, Nilsson B (1997). *p*CEA canalicular immunostaining in fine needle aspiration biopsy diagnosis of hepatocellular carcinoma. Acta Cytol.

[B90] Borscheri N, Roessner A, Rocken C (2001). Canalicular immunostaining of neprilysin (CD10) as a diagnostic marker for hepatocellular carcinomas. Am J Surg Pathol.

[B91] Lin F, Abdallah H, Meschter S (2004). Diagnostic utility of CD10 in differentiating carcinoma from metastatic carcinoma in fine-needle aspiration biopsy (FNAB) of the liver. Diagn Cytopathol.

[B92] Morrison C, Marsh W, Frankel WL (2002). A comparison of CD10 to *p*CEA, MOC-31, and Hepatocyte for the distinction of malignant tumors in the liver. Mod Pathol.

[B93] Chu PG, Ishizawa S, Wu E, Weiss LM (2002). Hepatocyte antigen as a marker of hepatocellular carcinoma: an immunohistochemical comparison to carcinoembryonic antigen, CD 10, and alpha-fetoprotein. Am J Surg Pathol.

[B94] Fan Z, van de Rijn M, Montgomery K, Rouse RV (2003). Hep Par 1 antibody stain for the differential diagnosis of hepatocellular carcinoma: 676 tumors tested using tissue microarrays and conventional tissue sections. Mod Pathol.

[B95] Siddiqui MT, Saboorian MH, Gokaslan ST, Ashfaq R (2001). Diagnostic utility of the HepPar1 antibody to differentiate hepatocellular carcinoma from metastatic carcinoma in fine-needle aspiration samples. Cancer (Cancer Cytopathol).

[B96] Zimmerman RL, Burke MA, Young NA, Solomides CC, Bibbo M (2001). Diagnostic value of hepatocyte paraffin 1 antibody to discriminate hepatocellular carcinoma from metastatic carcinoma in fine needle aspiration biopsies of the liver. Cancer (Cancer Cytopathol).

[B97] Leong AS-Y, Sormunen RT, Tsui WMS, Liew CT (1998). Hep Par 1 and selected antibodies in the immunohistological distinction of hepatocellular carcinoma from cholangiocarcinoma, combined tumours and metastatic carcinoma. Histopathology.

[B98] Maeda T, Kajiyama K, Adachi E, Takenaka K, Sugimachi K, Tsuneyoshi M (1996). The expression of cytokeratins 7, 19, and 20 in primary and metastatic carcinomas of the liver. Mod Pathol.

[B99] de Boer WB, Segal A, Frost FA, Sterrett GF (2000). Can CD 34 discriminate between benign and malignant hepatocytic lesions in fine-needle aspirates and thin core biopsies?. Cancer (Cancer Cytopathol).

[B100] Kong CS, Appenzeller M, Ferrell LD (2000). Utility of CD 34 reactivity in evaluating focal nodular hepatocellular lesions sampled by fine needle aspiration biopsy. Acta Cytol.

